# Risk factors for pneumonitis in advanced extrapulmonary cancer patients treated with immune checkpoint inhibitors

**DOI:** 10.1186/s12885-022-09642-w

**Published:** 2022-05-16

**Authors:** Yasuki Uchida, Daisuke Kinose, Yukihiro Nagatani, Sachiko Tanaka-Mizuno, Hiroaki Nakagawa, Kentaro Fukunaga, Masafumi Yamaguchi, Yasutaka Nakano

**Affiliations:** 1 Division of Respiratory Medicine, Department of Internal Medicine, Shiga University of Medical Science, Otsu, Japan; 2 Department of Radiology, Shiga University of Medical Science, Otsu, Japan; 3 Department of Digital Health and Epidemiology, Kyoto University, Kyoto, Japan; 4 The Center for Data Science Education and Research, Shiga University, Hikone, Japan; 5 Department of Respiratory Medicine, Kohka Public Hospital, Kohka, Japan

**Keywords:** Extrapulmonary tumor, Immune checkpoint inhibitors, Lung metastasis, Nivolumab, Pembrolizumab, Pneumonitis

## Abstract

**Background:**

Immune-mediated pneumonitis has a high mortality rate; however, information regarding the related risk factors remains limited. This study aimed to analyze risk factors for pneumonitis, including smoking and lung metastasis (LM), in patients with extrapulmonary primary tumors.

**Methods:**

Data of 110 patients treated with immune checkpoint inhibitors (ICIs) (nivolumab/pembrolizumab) for treating extrapulmonary primary tumors at the Shiga University of Medical Science Hospital between January 2015 and December 2019 were retrospectively collected. The association between the onset of pneumonitis and treatment-related factors was analyzed by logistic regression. The severity of pneumonitis was graded according to the Common Terminology Criteria for Adverse Events version 5.0. Risk factors, such as the absence or presence of interstitial lung disease (ILD) and LM, or other clinical factors, including smoking status before ICI administration, were analyzed.

**Results:**

Multivariate analyses indicated that the amount of smoking was significantly associated with an increase in the development of all-grade pneumonitis types (odds ratio (OR) = 20.33, 95% confidence interval (CI) = 20.03–20.66; *p* = 0.029). LM and ILD were significantly related to an increase in the development of symptomatic pneumonitis (≥ Grade 2) (OR = 10.08, 95% CI = 1.69–199.81; *p* = 0.076, and OR = 6.76, 95% CI = 1.13–40.63; *p* = 0.037, respectively).

**Conclusions:**

Pre-screening for ILD and LM and recognizing patients’ smoking history is important for determining the risk of ICI-induced pneumonitis and allowing safe ICI administration.

## Background

Immune checkpoint inhibitors (ICIs) are currently the first choice of treatment for several advanced carcinomas [1]. However, with the increasing number of patients receiving ICIs, the incidence of immune-related adverse events (ir-AEs) has also risen. There are various ir-AEs, and their profiles vary depending on the cancer type rather than on the ICI drugs used. AEs in areas located close to the primary lesions tend to occur as ir-AEs. For example, the incidence rate of skin rash during nivolumab (NIVO) therapy is 15% in malignant melanoma, which is higher than that associated with other malignancies, such as non-squamous non-small cell lung cancer (9%), gastric or gastro-esophageal junction cancer (7%), and esophageal squamous cell carcinoma (11%) [2–5]. Skin rash incidence during pembrolizumab (PEM) treatment is also higher in melanoma than in other malignancies [6–9]. Diarrhea frequency in association with NIVO is 21% in DNA mismatch repair-deficient or microsatellite instability-high colorectal cancer, while it is 8% in non-squamous non-small cell lung cancer, 16% in melanoma, and 7% in gastric or gastro-esophageal junction cancer [2–4, 10]. Pneumonitis is a potentially serious complication associated with checkpoint inhibitors, defined as focal or diffuse inflammation of the lung parenchyma. Asymptomatic patients can be treated by drug cessation alone or may be acceptable for follow-up, whereas symptomatic patients need the administration of glucocorticoids, including prednisone, with close follow-up and drug cessation. Additional immunosuppression, including infliximab or cyclophosphamide or intravenous immunoglobulin and mycophenolate mofetil, may be needed if the patient’s condition worsens, although the benefit from these treatments is unclear. Even after recovery, it is recommended that patients with moderate or severe disease should not resume treatment. Hypoxia may occur and progress rapidly, leading to death [11–13]. As reported based on the univariate generalized estimating equation model for programmed cell death 1 inhibitor-related pneumonitis, non-small cell lung cancer is related to a significantly higher incidence of pneumonitis than melanoma for all-grade pneumonitis (4.1% vs. 1.6%; *p* = 0.002). Comparing the incidence of pneumonitis, non-small cell lung cancer does not show incidence rates significantly different from those of renal cell carcinoma [14]. Both carcinomas have been previously implicated in smoking and carcinogenesis; thus, smoking-related cancers, such as lung cancer and renal cell carcinoma, may be linked to pneumonitis. Patients with lung cancer are more likely to be affected by smoking, and the lesions are located in the lungs; as a result, the frequency of pneumonitis is higher. However, the risk of developing pneumonitis from smoking and lung lesions in patients with other types of cancer, except lung cancer, is unknown. Other than the presence of interstitial lung disease (ILD) [15–18] and differences in carcinoma, the risk factors for pneumonitis are unclear. Therefore, we analyzed risk factors, including smoking and lung metastasis (LM), for pneumonitis in extrapulmonary primary tumors. To determine the risk of pneumonitis, the risk of developing all grades of pneumonitis, including asymptomatic pneumonitis, was examined. We also focused on pneumonitis with symptoms as we suspected this was clinically important. Asymptomatic pneumonitis can only be followed up, whereas symptomatic pneumonitis requires treatment, including steroid administration, and can progress to a more severe condition if left untreated. In this study, identifying risk factors for immune checkpoint inhibitor-induced pneumonitis is expected to help clarify matters that should be checked before administering immune checkpoint inhibitors.

## Methods

### Study design and participants

Data of 110 patients with advanced head and neck cancer, gastric cancer, renal cell cancer, urothelial cancer, peritoneal mesothelioma, and melanoma who were treated with programmed cell death 1 inhibitor (NIVO or PEM) at the Shiga University of Medical Science Hospital between January 2015 and December 2019 were retrospectively collected. To analyze the effect of LM, cancers originating from the lung fields, such as lung cancer, pleural mesothelioma, and primary pulmonary melanoma, were excluded. LM included pleural lesions with or without pleural effusions, but not pleural effusions alone with no histological evidence of disease to exclude pleural effusions of other causes. The number and distribution of pulmonary metastases were not considered, but their presence or absence was assessed. Patients simultaneously treated with other cancer therapies involving cytotoxic agents, except ICIs, were excluded. The patients treated with sequential cytotoxic/targeted therapy were included. All patients received the first ICI regimen, and patients who re-challenged ICI were excluded. This retrospective study was approved by the Institutional Review Board of Shiga University of Medical Science (R2020-037), which waived the need for written informed consent from the patients owing to the retrospective study design. 

### Medical record review

The severity of pneumonitis was graded according to the Common Terminology Criteria for Adverse Events version 5.0. The diagnosis of pneumonitis was established, and the evaluation of its severity was performed based on the patients’ clinical symptoms, physical examination findings, and radiographic images through a medical record review. The diagnostic criteria for pneumonitis were as follows: (1) new consolidation or ground-glass abnormality on both or any sides on chest computed tomography (CT) during treatment with ICI; (2) exclusion of pulmonary embolism using an electrocardiogram, laboratory data, contrast-enhanced CT, and echocardiography; (3) exclusion of pulmonary infection (i.e., pneumonitis that does not improve even after antibiotic therapy, or when cultures of sputum or bronchoalveolar lavage fluid are free of bacteria); (4) exclusion of heart failure using an electrocardiogram, laboratory data, and echocardiography (asymptomatic patients without CT confirmation were excluded); and (5) exclusion of tumor progression using laboratory data and/or biopsy. In each case of pneumonitis, the pattern of pneumonitis was classified according to the American Thoracic Society/European Respiratory Society statement [19] into (i) organizing pneumonia (OP) pattern, (ii) nonspecific c (NSIP) pattern, (iii) usual interstitial pneumonia pattern, (iv) hypersensitivity pneumonitis pattern, (v) acute interstitial pneumonia/acute respiratory distress syndrome pattern, and (vi) unclassifiable patterns, with reference to preceding studies [20, 21]. All chest CT findings were reviewed by the consensus of a pulmonologist (Yasuki Uchida) and a radiologist (Yukihiro Nagatani). Preexisting ILD was defined using the criteria for ILD of the European Respiratory Society/American Thoracic Society [19]. Other clinical data were collected through a medical record review. The time point for evaluations of smoking status was at the time of ICI initiation. Ever smokers were defined as current and former smokers. Never smokers were defined as those who have never smoked. We evaluated the amount of smoking as pack years.

### Statistical analysis

The characteristics and treatment-related factors of patients with and without pneumonitis or symptomatic and asymptomatic were compared using the Mann–Whitney *U* test or Fisher’s exact test, as appropriate. Odds ratios (ORs) were estimated using logistic regression analysis. All *p*-values were two-sided, and those ≤ 0.05 were considered statistically significant. Predictors with *p*-values < 0.10 in the univariate analysis were included in a multivariate model. ILD is the most common consensus clinical parameter related to the development of pneumonitis [15–18]. We selected ILD from previous studies and also selected the parameters from the results of univariate analysis and performed multivariate analysis. If multiple parameters fulfilled the conditions, the parameter with a *p*-value < 0.05 or the smallest *p*-value was included in the multivariate analysis. Moreover, if several smoking-related parameters met the conditions, the amount of smoking was included in the model as it was more specific and related. Two-sided Cohen’s κ-coefficients were used to calculate the level of agreement in terms of the pneumonitis pattern in the radiologic assessment between the pulmonologist and the radiologist. All statistical analyses were performed using JMP version 11 (SAS Institute Inc., Cary, NC, USA), R version 3.5.1 (R Foundation for Statistical Computing, Vienna, Austria) [22], and EZR version 1.50 on R commander (Saitama Medical Center, Jichi Medical University, Saitama, Japan) [23].

## Results

### Patient characteristics

The mean duration of pneumonitis onset was 5.6 months, and the mean follow-up period for patients without pneumonitis was 13.3 months. There were 18, 43, 31, 10, 5, 2, and 1 patients with first-line, second-line, third-line, fourth-line, fifth-line, sixth-line, and seventh-line ICI therapy, respectively.

The results of the comparison of the clinical characteristics comparing patients without pneumonitis, asymptomatic (Grade 1) pneumonitis patients and symptomatic (≥ grade 2) patients are shown in Table 1. Comparison between patients with pneumonitis and those without pneumonitis showed significant differences in the amount of smoking (*p* = 0.024) and lung metastasis (*p* = 0.029). Two patients (one with head and neck cancer and the other with urothelial cancer) had received radiotherapy prior to ICI administration, but they did not develop pneumonitis.

**Table 1:** Patient and treatment characteristics

**Patient characteristics**	**All patients** ***n*** = 110	**No pneumonitis patients** ***n*** = **91**		**Pneumonitis patients** **n = 19**	***p*-value** **No pneumonitis vs pneumonitis (≥ grade 1)**
Age					0.44
Median (range)	68 (34–84)	67 (34–84)		69 (53–81)	
Sex					0.14
Male	82	65		17	
Female	28	26		2	
Smoking history					0.10
Current	25	19		6	
Former	45	35		10	
Never	40	37		3	
Amount of smoking (pack-years)					0.024
Median (range)	21.75 (0–190)	15.00 (0–98)		36.75 (0–190)	
Types of cancer					0.40
Head and neck cancer	23	17		6	
Gastric cancer	17	14		3	
Renal cell cancer	29	23		6	
Urothelial cancer	15	12		3	
Malignant melanoma	25	24		1	
Peritoneal mesothelioma	1	1		0	
BMI (kg/m^2^)					0.67
Median (range)	20.37 (14.61–36.49)	20.56 (14.63–36.49)		20.08 (14.61–32.36)	
Immunotherapy					0.41
NIVO	95	79		16	
PEM	15	12		3	
ILD					0.068
Yes	10	6		4	
No	100	85		15	
Lung metastasis					0.31
Yes	69	55		14	
No	41	36		5	
Treatment line					0.66
Median (range)	2 (1–7)	2 (1–7)		3 (1–5)	
TKI					1.00
Yes	28	23		5	
No	82	68		14	
CRP (mg/dl)					0.82
Median (range)	0.98 (0.01–11.97)	0.98 (0.01–11.97)		0.79 (0.08–10.49)	
NLR					0.33
Median (range)	3.84 (0.77–39.78)	3.79 (1.00–39.78)		4.46 (0.77–11.36)	
**Patient characteristics**	**Asymptomatic (Grade 1) pneumonitis patients** ***n*** = **7**		**Symptomatic (≥ grade 2) pneumonitis patients** ***n*** = **12**		***p*-value** **Asymtomatic (≤ grade 1) vs Symptomatic (≥ grade 2)**
Age					0.72
Median (range)	73 (58–81)		68.5 (53–74)		
Sex					0.28
Male	6		11		
Female	1		1		
Smoking history					0.31
Current	2		4		
Former	4		6		
Never	1		2		
Amount of smoking (pack-years)					0.057
Median (range)	36.00 (0–67.5)		40.88 (0–190)		
Types of cancer					0.090
Head and neck cancer	4		1		
Gastric cancer	2		2		
Renal cell cancer	0		6		
Urothelial cancer	0		3		
Malignant melanoma	1		0		
Peritoneal mesothelioma	0		0		
BMI (kg/m^2^)					0.69
Median (range)	20.16 (17.63–25.68)		20.04 (14.61–32.36)		
Immunotherapy					0.21
NIVO	7		9		
PEM	0		3		
ILD					0.077
Yes	1		3		
No	6		9		
Lung metastasis					0.029
Yes	3		11		
No	4		1		
Treatment line					0.073
Median (range)	2 (1–3)		2 (3–5)		
TKI					0.18
Yes	0		5		
No	7		7		
CRP (mg/dl)					0.71
Median (range)	0.47 (0.25–10.49)		1.16 (0.08–9.7)		
NLR					0.35
Median (range)	3.69 (0.77–11.36)		4.80 (2.35–10.42)		

*BMI* Body mass index, *NIVO* Nivolumab, *PEM* Pembrolizumab, *ILD* Interstitial lung disease, *LM* Lung metastasis, TKI Tyrosine kinase inhibitor, NLR Neutrophil-to-lymphocyte ratio

Among 110 patients who received ICIs, 19 (17.2%) developed pneumonitis. Table 2 presents clinical details of the 19 patients who developed pneumonitis, and Table 3 shows summaries for 19 people. The OP pattern was most commonly observed (9 out of 19; 47.3%), followed by the NSIP pattern (6 out of 19; 31.6%). Three patients (15.8%) had concomitant NSIP and OP patterns. Hypersensitivity pneumonitis patterns were found in one patient (5.3%). Cohen’s κ-coefficient was 0.676.

**Table 2:** Clinical characteristics of 19 patients with pneumonitis

Pt	Tumor	sex	Age, year	Agents	ILD	LM	Smoking history	Smoking amount, pack-years	Time to the onset of pneumonitis, day	Grade	Radiographic pattern
1	HNC (Sq)	M	73	NIVO	−	+	F	67.5	112	1	OP + NSIP
2	HNC (Sq)	M	79	NIVO	+	−	F	57	42	1	OP + NSIP
3	HNC (Undifferentiated)	M	68	NIVO	+	+	F	48	87	2	NSIP
4	HNC (Sq)	po	66	NIVO	−	+	C	22.5	59	1	OP + NSIP
5	HNC (Sq)	M	71	NIVO	−	−	F	40	6	1	OP
6	HNC (Sq)	M	69	NIVO	−	+	C	50	111	5	NSIP
7	MM	F	78	NIVO	−	−	N	0	105	1	OP
8	GC (AdSq)	M	72	NIVO	−	+	C	26.5	456	2	NSIP
9	GC (Ad)	M	58	NIVO	−	+	C	36	20	1	OP
10	GC (Ad)	M	81	NIVO	−	+	F	15	287	1	HP
11	RCC (clear cell)	M	74	NIVO	−	+	F	36.75	771	2	NSIP
12	RCC (clear cell)	M	54	NIVO	−	+	C	16	371	2	OP
13	RCC (clear cell)	M	63	NIVO	−	−	F	64.5	105	2	OP
14	RCC (clear cell)	F	64	NIVO	−	+	N	0	25	2	OP
15	RCC (clear cell)	M	73	NIVO	+	+	F	22.5	129	2	OP
16	RCC (clear cell)	M	53	NIVO	−	+	N	0	1068	2	NSIP
17	UC	M	69	PEM	+	+	C	12	2	5	NSIP
18	UC	M	64	PEM	−	+	F	102	343	3	OP
19	UC	M	69	PEM	−	+	F	190	229	2	OP

*Ad* Adenocarcinoma, *C* Current, *F* Female, *F* Former, *GC* Gastric cancer, *HNC* Head and neck cancer, *HP* hypersensitivity pneumonitis, *ILD* Interstitial lung disease, *LM* Lung metastasis, *M* male, *MM* Malignant melanoma, *N* Never, *NIVO* Nivolumab, *NSIP* Nonspecific interstitial pneumonia, *OP* Organizing pneumonia, *PEM* Pembrolizumab, *RCC* Renal cell carcinoma, *Sq* Squamous carcinoma, *UC* Urothelial carcinoma

**Table 3:** Summary of characteristics of patients with pneumonitis

Patient characteristics	
Age, Median (range)	69 (53–81)
Sex, Male/Female	17/2
Smoking history Current/Former/Never	6/10/3
Amount of smoking (pack-years) Median (range)	36.75 (0–190)
Tumor HNC/MM/GC/RCC/UC	6/1/3/3/6/3
BMI(kg/m^2^), Median (range)	20.08 (14.61–32.326)
Immunotherapy, NIVO/PEM	16/3
ILD, Yes/No	4/15
LM, Median (range)	14/5
Treatment line, Median (range)	3 (1–5)
TKI, Yes/No	5/14
CRP(mg/dl), Median (range)	0.79 (0.08–10.49)
NLR, Median (range)	4.46 (0.77–11.36)
OP/NSIP/OP + NSIP/HP	9/6/3/1

*C* Current, BMI Body mass index, *F* Female, *F* former, *GC* Gastric cancer, *HNC* Head and neck cancer, *HP* Hypersensitivity pneumonitis, *ILD* Interstitial lung disease, *LM* Lung metastasis, *M* Male, *MM* Malignant melanoma, *N* Never, NLR Neutrophil-to-lymphocyte ratio, *NIVO* Nivolumab, *NSIP* nonspecific interstitial pneumonia, *OP* Organizing pneumonia, *PEM* Pembrolizumab, *RCC* Renal cell carcinoma, TKI Tyrosine kinase inhibitor, *UC* Urothelial carcinoma

The median duration between treatment initiation and the occurrence of asymptomatic (G1) and symptomatic (≥ G2) pneumonitis was 3.75 months (range: 0.1–38.1) and 8.0 months (range: 0.2–77.8), respectively.

### Incidence of pneumonitis

Table 4 shows the logistic regression analysis results for the incidence of all-grade and symptomatic pneumonitis. The amount of smoking (OR = 20.35 per 20-pack year increase, 95% CI = 20.06–20.68; *p* = 0.018) and smoking history (ever smoker vs. never smoker [OR = 3.49, 95% CI = 1.06–15.77; *p* = 0.038]) were associated with all-grade pneumonitis. LM (OR = 7.58, 95% CI = 1.39–141.38; *p* = 0.015) and the amount of smoking (OR = 20.39 per 20-pack-year increase, 95% CI = 20.06–20.76; *p* = 0.020) were significantly associated with an increase in the development of symptomatic pneumonitis. In the multivariate analysis, LM was included for symptomatic pneumonitis based on the p-value. The amount of smoking was included for all-grade pneumonitis with ILD. The amount of smoking was included for symptomatic pneumonitis with ILD. Multivariate analyses indicated that the amount of smoking was significantly associated with an increase in the development of all-grade pneumonitis (OR = 20.33, 95% CI = 20.03–20.66; *p* = 0.029), and LM was significantly related to an increase in the development of symptomatic pneumonitis (OR = 10.08, 95% CI = 1.69–199.81; *p* = 0.076). In addition, ILD was significantly associated with an increase in the development of symptomatic pneumonitis in the multivariate analysis (6.76, 95% CI = 1.13–40.63; *p* = 0.037).

**Table 4:** Analyses of the incidence of pneumonitis using logistic regression models

Factor	Symptomatic (≥ G2) pneumonitis				All-grade (≥ G1) pneumonitis			
	Univariate Analysis		Multivariate Analysis		Univariate Analysis		Multivariate Analysis	
	OR (95% CI)	*P*-values	HR (95% CI)	*P*-values	OR (95% CI)	*P*-values	HR (95% CI)	*P*-values
LM		0.015		0.0076		0.26		
+ vs −	7.58 (1.39–141.38)		10.08 (1.69–199.81)		1.83 (0.64–6.39)			
Age		0.99				0.30		
per 1-year increase	0.99 (0.95–1.06)				1.02 (0.98–1.08)			
Sex		0.11				0.08		
Male vs Female	4.18 (0.76–78.27)				3.40 (0.89–22.40)			
Smoking history								
Ever vs Never	3.03 (0.75–20.44)	0.13			3.49 (1.06–15.77)	0.038		
Amount of Smoking (pack-years)		0.020				0.018		0.029
per 20-pack year increase	20.39 (20.06–20.76)				20.35 (20.06–20.68)		20.33 (20.03–20.66)	
BMI (kg/m^2^)		0.90				0.99		
per 1 kg/m^2^	1.01 (0.87–1.16)				1.00 (0.88–1.12)			
ILD		0.071		0.037		0.078		0.12
Yes vs No	3.78 (0.88–14.90)		6.76 (1.13–40.63)		4.33 (0.83–18.88)		3.19 (0.72–13.01)	
Treatment line		0.19				0.92		
	1.36 (0.85–2.12)				1.02 (0.66–1.51)			
TKI		0.19				0.92		
Yes vs No	2.33 (0.64–8.01)				1.06 (0.31–3.11)			
CRP (mg/dl)		0.69				0.84		
	1.04 (0.84–1.23)				1.02 (1.18–0.98)			
NLR		0.97				0.94		
	1.00 (0.83–1.11)				1.00 (0.88–1.10)			

*OR Odds ratio, CRP C-reactive protein, CI* Confidence interval, *BMI* Body mass index, *HR* Hazard ratio, *ILD* Interstitial lung disease, *LM* Lung metastasis, *TKI* Tyrosine kinase inhibitor, *NLR* Neutrophil-to-lymphocyte ratio

## Discussion

This study yielded two major findings. First, the amount of smoking was identified as a significant risk factor for the onset of both symptomatic and all-grade pneumonitis. Second, LM was a significant risk factor for developing symptomatic pneumonitis.

For every 20-pack-year increase in the amount of smoking, the risk of symptomatic or all-grade pneumonitis increased by 20.3 times. Current and former smokers had a significantly higher risk of pneumonitis than never smokers. To the best of our knowledge, this study is the first to show that the smoking index is an independent risk factor for ICI-related pneumonitis in extrapulmonary cancer patients. Naidoo et al. showed that the incidence of pneumonitis, including that in lung cancer, was not significantly different between never smokers (19 out of 43 [44%]) and both former/current smokers (24 out of 43 [56%]); however, current smokers showed a tendency toward higher incidence rates than those of former smokers (5 out of 23 vs. 0 out of 19, *p* = 0.053) [24]. This study did not examine the amount of smoking. Therefore, the results differed slightly from those of our study, but they tend to be consistent with the results of our study. Delaunay et al. reported that most patients who developed pneumonitis were either current (26.7%) or former (53.3%) smokers, with a median consumption of 40 (5–80) pack-years [21]. Our patients had multiple cancer types, although most were non-small cell lung cancers. The smoking index is an independent risk factor for acute exacerbation in patients with cancer complicated by interstitial pneumonia who are receiving cytotoxic anticancer drugs [25]. In terms of other pulmonary diseases, smoking is a risk factor in idiopathic pulmonary fibrosis [26, 27]; however, there is currently a lack of clarity on the involvement of smoking and coexisting emphysema in acute exacerbation events [28–31]. In this study, pre-treatment CT was not taken under the same conditions; thus, the effects of the extent of emphysematous lesions could not be assessed. Concerning radiation pneumonitis, the existing data on smoking and comorbid emphysema as risk factors are inconsistent [32–35]. Conversely, interstitial pneumonia was correlated with symptomatic pneumonitis in this study. Similarly, some studies identified ILD as a risk factor for immune-mediated pneumonitis [15–18]. ILD is also a risk factor in radiation pneumonitis [36, 37] and other anticancer drug-related pneumonitis cases [38, 39], which should be noted in pneumonitis. Nishino et al. compared the incidence of pneumonitis in association with PD-1 inhibitor use across different tumor types and reported that the incidence in non-small cell lung cancer was significantly higher in both all-grade (4.1% vs. 1.6%; *p* = 0.002) and ≥ grade 3 (1.8% vs. 0.2%; *p* < 0.001) diseases than in melanoma [14]. If the lesion is located in the chest, such as in lung cancer, the risk of symptomatic pneumonitis may increase. Therefore, symptomatic pneumonitis requires attention regarding the administration of ICIs for cancers that originate outside the chest with pulmonary metastases. To the best of our knowledge, this is the first study to demonstrate a relationship between symptomatic immune-mediated pneumonitis and LM. As the population was limited in this study and the analysis of the relationship between smoking and LM was not the primary endpoint, it is necessary to validate our findings in a larger number of patients independent of anticancer drug use. Recently, ICI use has been suggested to be associated with the severity of coronavirus disease (COVID-19) and the related rate of hospitalization [40]. Therefore, the presence of viral infection may also need to be examined as a risk factor for pneumonitis. None of the patients in this study had COVID-19, and the disease was still not endemic in Japan at the end of this study.

The diagnosis of drug-induced pneumonitis types, such as immune-mediated pneumonitis, is difficult to establish based on imaging alone and must be considered in conjunction with other examinations, such as clinical history, pulmonary function tests, cardiac evaluation, histopathology, and bronchoscopy. Therefore, the quality of this type of study and patient care would be better if radiologists and respiratory physicians who care for the patient in clinical practice work together. In fact, most of the patients with pneumonitis in this study consulted respiratory physicians and were diagnosed through bronchoscopy or other means.

The frequency of pneumonitis varies from 2.7% to 16.9% across reports [14, 16–18, 20, 21, 24, 41]. The incidence of pneumonitis is higher in the Japanese population (13.2–16.9%) [16–18, 41] than in the non-Japanese population (3.5–11.8%) [20, 21, 24]. The Japanese population also tends to have a high incidence of pneumonitis induced using epidermal growth factor receptor tyrosine kinase inhibitors [38, 42]. It is unclear whether this can be attributed to race or the presence of confounding factors unique to Japan, such as the prevalence of interstitial pneumonia or the amount of smoking. Alternately, the fact that there is a tendency to repeat imaging studies in Japan may have facilitated the detection of grade 1 asymptomatic pneumonitis. If the prevalence of pneumonitis is high in the Japanese population and low in the non-Japanese population, it may be easier to investigate risk factors for pneumonitis in Japanese people who are more likely to experience a higher rate of events. It should also be considered that the incidence varies with the follow-up duration.

This study had some limitations. First, it had a retrospective design and included a relatively small number of patients with immune-mediated pneumonitis treated in a single institution. This could be a possible reason for the wide 95% CIs for several factors. The association with grade 3 or higher pneumonitis was too small to analyze. Second, data on asymptomatic pneumonitis during the follow-up of patients with cancers with extrapulmonary origins might have been missed owing to a lack of routine chest imaging evaluations, such as chest radiography. Third, sequential therapy may have influenced the results; however, none of the patients had received EGFR inhibitors. Finally, several performance statuses and pulmonary function parameter values were missing and could not be analyzed. Therefore, validation in a multicenter, prospective study that considers these points is necessary.

## Conclusions

In this study, which aimed to examine the predictors of pneumonitis development risk in association with the administration of ICIs for extrapulmonary tumors, the smoking index was found to increase the risk of pneumonitis regardless of the past or current smoking status. Similarly, interstitial pneumonitis and LM also increased the risk of symptomatic pneumonitis development. Pre-screening for ILD and LM and recording patients’ smoking history can aid in the determination of the risk of pneumonitis and ensure safe ICI administration. It is our sincere hope that our findings can be used for the successful management of immune-mediated pneumonitis to increase the life expectancy of cancer patients.

## Data Availability

The datasets used and/or analyzed during the current study are available from the corresponding author on reasonable request.

## Abbreviations

ICI: Immune checkpoint inhibitor
ir-AE: Immune-related adverse event
NIVO: Nivolumab
PEM: Pembrolizumab
ILD: Interstitial lung disease
LM: Lung metastasis
OP: Organizing pneumonia
NSIP: Nonspecific interstitial pneumonia
